# Adaptation to Human Populations Is Revealed by Within-Host Polymorphisms in HIV-1 and Hepatitis C Virus

**DOI:** 10.1371/journal.ppat.0030045

**Published:** 2007-03-30

**Authors:** Art F. Y Poon, Sergei L. Kosakovsky Pond, Phil Bennett, Douglas D Richman, Andrew J. Leigh Brown, Simon D. W Frost

**Affiliations:** 1 Department of Pathology, University of California San Diego, La Jolla, California, United States of America; 2 Science Park, University of Warwick, Coventry, United Kingdom; 5 Institute of Evolutionary Biology, University of Edinburgh, Edinburgh, Scotland, United Kingdom; National Institute of Allergy and Infectious Diseases, United States of America; 3 School of Medicine, University of California San Diego, La Jolla, California, United States of America; 4 Veterans Affairs San Diego Healthcare System, San Diego, California, United States of America

## Abstract

CD8^+^ cytotoxic T-lymphocytes (CTLs) perform a critical role in the immune control of viral infections, including those caused by human immunodeficiency virus type 1 (HIV-1) and hepatitis C virus (HCV). As a result, genetic variation at CTL epitopes is strongly influenced by host-specific selection for either escape from the immune response, or reversion due to the replicative costs of escape mutations in the absence of CTL recognition. Under strong CTL-mediated selection, codon positions within epitopes may immediately “toggle” in response to each host, such that genetic variation in the circulating virus population is shaped by rapid adaptation to immune variation in the host population. However, this hypothesis neglects the substantial genetic variation that accumulates in virus populations within hosts. Here, we evaluate this quantity for a large number of HIV-1– (*n* ≥ 3,000) and HCV-infected patients (*n* ≥ 2,600) by screening bulk RT-PCR sequences for sequencing “mixtures” (i.e., ambiguous nucleotides), which act as site-specific markers of genetic variation within each host. We find that nonsynonymous mixtures are abundant and significantly associated with codon positions under host-specific CTL selection, which should deplete within-host variation by driving the fixation of the favored variant. Using a simple model, we demonstrate that this apparently contradictory outcome can be explained by the transmission of unfavorable variants to new hosts before they are removed by selection, which occurs more frequently when selection and transmission occur on similar time scales. Consequently, the circulating virus population is shaped by the transmission rate and the disparity in selection intensities for escape or reversion as much as it is shaped by the immune diversity of the host population, with potentially serious implications for vaccine design.

## Author Summary

The rapid accumulation of genetic variation in human viruses, such as human immunodeficiency virus type 1 (HIV-1) and hepatitis C virus (HCV), enables these pathogens to elude the immune system and forestalls the development of effective vaccines. This variation may be shaped by selection due to host-specific immune responses, such that the total virus population mirrors the immune diversity of the host population. However, the often-neglected viral genetic variation within hosts may also play an important role in shaping variation in the total virus population. We carry out an innovative analysis of bulk HIV-1 and HCV sequences isolated from over 4,000 human patients, exploiting “mixtures” (i.e., ambiguous nucleotides) as a site-specific marker of within-host genetic variation. We find that nonsynonymous mixtures are disproportionately abundant at codon positions under strong host-specific immune selection. Because existing models of virus evolution provide no explanation for this outcome, we have formulated a new model supplemented with stochastic simulations to demonstrate that the rapid transmission of viruses through diverse selective environments creates a positive correlation between nonsynonymous variation within and among hosts.

## Introduction

The cellular immune response mediated by CD8^+^ cytotoxic T-lymphocytes (CTLs) performs a critical role in the immune control of human viruses such as human immunodeficiency virus (HIV-1) [1] and hepatitis C virus (HCV) [2]. Consequently, the major histocompatibility (MHC) loci that encode the human leukocyte antigen (HLA) class I molecules, which recognize and bind CTL epitopes in viral proteins, are among the most highly polymorphic genes in the human population [3]. Nevertheless, the CTL response often fails to control the infection completely because of mutations that occur within HLA-restricted CTL epitopes, enabling the virus to escape binding and recognition [4]. Because epitopes are often located in functionally conserved regions of the viral genome, escape mutations may become costly to maintain in the absence of a selective HLA allele [5,6]. Thus, when an escape variant is transmitted between HLA-mismatched individuals, competitive growth frequently selects for reversion of the mutation to wild-type, as demonstrated experimentally in simian immunodeficiency virus–infected rhesus macaques [7] and in a comparative study of HIV-1–infected human patients [8].

Consequently, host-specific selection for escape or reversion may play an important role in shaping genetic variation in the circulating virus population [1,2,5,9,10]. For instance, population-based analyses of HIV-1 [9] and HCV [11] sequences have found several significant associations between divergent sites within CTL epitopes and the selective HLA alleles in the host population, suggesting that the frequency of escape polymorphisms in the circulating virus population are directly shaped by the immune diversity of the host population. Furthermore, the viral load of HIV-1–infected individuals has been found to be positively correlated with the frequency of the corresponding HLA supertypes in the host population, implying that the total virus population is adapting to the most frequent HLA supertypes [12]. If escape variants are readily transmitted between hosts, then a host with a common HLA supertype is more likely to encounter a virus that has already escaped its immune response [13], conferring a selective advantage to rare HLA supertypes. However, the virus genotype that becomes transmitted to the next host does not necessarily represent the ultimate outcome of adaptation to the previous host. Escape variants that have been transmitted into a host lacking a selective HLA allele can persist over long periods of time before reversion, or fail to revert at all over the duration of the study [8,14]. A delay or absence of reversion may be attributable to weak selection, when the fitness of the escape variant is either intrinsically high, or it has acquired compensatory mutations.

To evaluate the role of CTL-mediated selection in shaping the genetic variation of human viruses, we have carried out a large-scale analysis of HIV-1 and HCV protein-coding sequences isolated from human hosts. Previous analyses of clonal HIV-1 subtype B envelope [5,15] and protease (PR) [16] sequences have shown that across codon positions, genetic variation within hosts is positively correlated with variation among hosts. These correlations suggest that the genetic variation at both levels of the virus population is being shaped by a common set of biological constraints. However, the use of clonal sequences to characterize within-host variation restricted these analyses to small samples of hosts (*n* ≤ 12). In addition, quantifying the influence of selection on genetic variation within and among hosts is potentially confounded by variation in mutation rates among codon positions. Because mutation is the ultimate source of all genetic variation, site-specific variation at either level will be roughly proportional to the local mutation rate, which can yield a positive correlation in the absence of selection [17]. Indeed, this effect constitutes the basis for several tests of non-neutral evolution in genetic sequences [18–20].

To address the problem of limited sample size, we exploit “sequencing mixtures” as a site-specific marker of genetic variation within hosts. A sequencing mixture occurs when multiple distinct peaks occur above the same position in a sequencing electropherogram [21]; by convention, mixtures are encoded in sequences by ambiguous nucleotide characters (International Union of Pure and Applied Chemistry symbols “M”, “R”, “W”, “S”, “Y”, and “K”). Because mixtures can indicate the presence of a nucleotide polymorphism in the population, population-based (or “bulk”) sequencing is employed to detect minority variants that occur at frequencies above 10%–25% [21–23]. Although population-based sequencing may fail to detect mixtures below this threshold, transient polymorphisms under selection are more likely to be sampled at intermediate frequencies. This application of mixtures is particularly relevant to viruses with extremely high mutation rates such as HIV-1 and HCV, for which population-based sequences are exceedingly abundant. In this study, we use mixtures to quantify the effect of selection on within-host variation in population-based sequences of RT-PCR–amplified viral RNA from blood plasma isolated from over 4,000 HIV-1– or HCV-infected patients.

To remove the confounding effect of variation in mutation rates, we normalized the nonsynonymous variation per codon position by the synonymous variation, for either level of the virus population. Thus, we calculated the site-specific difference between the frequencies of nonsynonymous *(mN)* and synonymous mixtures *(mS),* and estimated the analogous difference between the rates of nonsynonymous *(dN)* and synonymous substitution *(dS).* Our estimates of *mN* and *dN* were both scaled by the expected number of nonsynonymous sites at each codon position; likewise, estimates *mS* and *dS* were scaled by the expected number of synonymous sites in the codon. The difference in substitution rates *(dN − dS)* is a conventional summary statistic for diversifying selection among hosts, i.e., host-specific selection causing nonsynonymous variation to accumulate among individual virus populations. We propose that the difference in mixture frequencies *(mN − mS)* can be employed as a summary statistic characterizing selection within each host. For instance, *mN − mS* > 0 can represent transient nonsynonymous polymorphisms undergoing directional selection (which drives the fixation of a specific variant within the host). Using these quantities, we will show that the distribution of mixtures in our samples of HIV-1 and HCV sequences cannot be explained by variation in mutation rates alone, and that host-specific selection is an important force shaping variation at both levels of the total virus population.

Because existing models of virus evolution seldom account for genetic variation both within and among hosts (but see [24,25]), we formulate a novel yet simple model that invokes both host-specific selection and rapid transmission between hosts to explain the observed patterns of genetic variation within and among hosts infected by HIV-1 or HCV. Bolstered by stochastic simulations, our model specifies the conditions that yield this outcome, and quantitatively predicts the joint effect of selection and transmission on the genetic composition of the circulating virus population. We find that when host-specific selection for escape and reversion is unbalanced and the transmission rate is high, then the frequency of escape variants becomes considerably skewed from expectations derived from the immune diversity of the host population. Failing to account for this effect may lead to erroneous conclusions on the overall importance of CTL-mediated selection in directing the evolution of the total virus population, or the relative contribution of specific CTL epitopes. Furthermore, the design of an effective vaccine to human viruses such as HIV-1 or HCV is highly contingent upon our ability to anticipate the response of an infection to CTL-mediated selection.

## Results

### Sequencing Mixtures Reveal CTL Selection

We screened for sequencing mixtures in population-based sequences of HIV-1 PR (*n* = 3,458) and reverse transcriptase (RT, *n* = 1,997) isolated from 3,004 and 1,989 treatment-naive individuals, respectively, and HCV sequences of envelope protein E1 (*n* = 2,691) and the hyper-variable region HVR1 of envelope protein E2 (*n* = 346). Although many sequences had at least one mixture (55% HIV-1, 63% HCV), there were relatively few mixtures per sequence on average (0.015 mixtures per codon position in HIV-1, 0.011 in HCV), suggesting that only a small number of codon positions had mixtures at detectable (20%–80%) frequencies in a given host (Figure S1). We found substantial variation among codon positions in mixture frequencies (Figure S2), which was greater for nonsynonymous (coefficient of variation = 1.98 HIV-1, 1.28 HCV) than synonymous mixtures (0.95 HIV-1, 1.06 HCV). There was no significant correlation between nonsynonymous and synonymous mixture frequencies per codon position in either HIV-1 (RT, Pearson's ρ = 0.04, *p*-value = 0.52; PR, ρ = 0.13, *p*-value = 0.21) or HCV gene sequences (E1, ρ = 0.01, *p*-value = 0.75; E2, ρ = −0.13, *p*-value = 0.18), indicating that the variation in mixture frequencies among codon positions was not simply due to local mutation rates.

**Figure 1:**
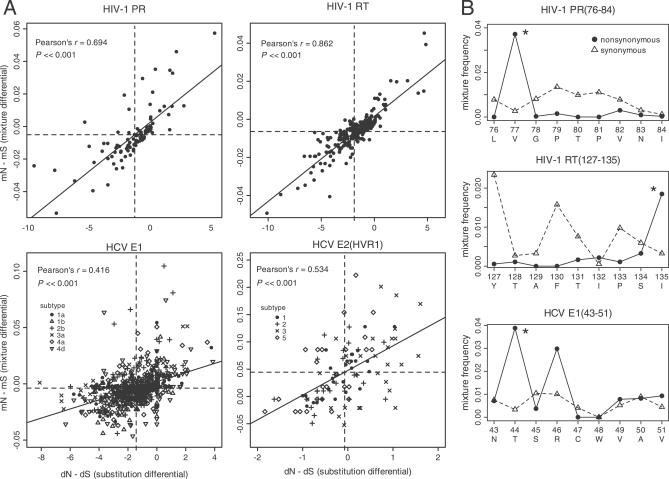
Genetic Variation within Hosts Is Shaped by Host-Specific Selection for CTL Escape (A) The difference in nonsynonymous and synonymous mixture frequencies within hosts *(mN* − *mS)* is positively correlated with diversifying selection among hosts *(dN* − *dS)* per codon position. Each point corresponds to a unique codon position in the respective gene sequence. Dashed lines indicate the mean value for each quantity, which is consistently negative in *dN* − *dS,* implying purifying selection overall. Solid lines indicate a linear fit to the data. HCV genotypes are plotted separately as shown in the figure legends. A single outlier caused by a rare substitution lies outside the plot region for HIV-1 RT, but does not influence the significance of this correlation (Pearson's ρ = 0.619, *p*-value < 3 × 10^−16^). (B) Selection for CTL escape elevates the frequency of nonsynonymous mixtures (solid circles) relative to synonymous mixtures (open triangles) at anchor residues within known A2-supertype–restricted epitopes in HIV-1 PR and RT and HCV E1 (predicted). Asterisks indicate anchor residues associated with disproportionately high frequencies of nonsynonymous mixtures.

The difference between nonsynonymous and synonymous mixture frequencies *(mN − mS)* was highly correlated with the difference between nonsynonymous and synonymous substitution rates *(dN − dS)* per codon position for both HIV-1 and HCV gene sequences (Figure 1A). This positive correlation between *dN* − *dS* and *mN − mS* remained significant for both E1 and E2 gene sequences even when different genotypes of HCV were analyzed separately. Overall, the quantity *dN − dS* assumed a negative value when averaged across the gene sequence, implying that nonsynonymous variation at the majority of codon positions was largely neutral or deleterious throughout the host population. Nevertheless, we detected significant diversifying selection (*dN − dS* > 0) at nine codon positions in HIV-1 PR (12, 13, 19, 35, 37, 63, 64, 77, and 93) and eight positions in RT (35, 39, 102, 122, 135, 200, 211, and 245) after correcting for the false-discovery rate [26] (α = 0.05); likewise, significant diversifying selection was attributed to several codon positions in HCV E1 and E2 (HVR1) sequences, which varied by genotype.

For specific CTL epitopes in HIV-1 PR, RT, and HCV E1 sequences, we observed disproportionately higher frequencies of nonsynonymous mixtures at the anchor residues (Figure 1B) critical for MHC binding. In contrast, the profile of synonymous mixture frequencies within these epitopes lacked any distinct peaks in association with anchor residues. Overall, the median difference between the frequencies of nonsynonymous and synonymous mixtures was significantly greater at known HLA-B–restricted epitopes (median *mN − mS* = −0.2% mixtures per sequence per site) than in the remainder of the HIV-1 RT sequence (−0.5%; Wilcoxon rank-sum test, *p*-value = 0.007). We also found that *mN − mS* was greater at the anchor residues of HLA-B–restricted epitopes (median = −0.2%) than in an equivalent random sample of codon positions from HIV-1 RT on average (median = −0.4%), but this difference was only marginally significant (*p*-value = 0.11). In contrast, the median was not significantly greater at the known HLA-A–restricted epitopes within RT (Wilcoxon rank-sum test, *p*-value = 0.22), consistent with previous studies suggesting that HLA-B alleles assume a dominant role in the CTL control of HIV-1 [9,27]. In HIV-1 PR, the median excess in nonsynonymous mixtures was considerably greater within the single known HLA-B–restricted epitope (median = 0.7%) than in the rest of the gene sequence (median = −0.4%), but this difference was only marginally significant due to the small sample of codon positions (Wilcoxon rank-sum test, *p*-value = 0.1). Again, there was no significant difference in median values between HLA-A–restricted epitopes and the remainder of the PR sequence (Wilcoxon rank-sum test, *p*-value = 0.55).

Similarly, in the HCV E1 sequences, we found that the median excess of nonsynonymous mixtures was significantly greater within the two known HLA-B–restricted epitopes (median = 0.9%) than in an equivalent random sample of codon positions (median = −0.2%; Wilcoxon rank-sum test, *p*-value = 0.023). However, the median value for known HLA-A–restricted epitopes in HCV E1 was significantly less (median = −0.5%) than that in the remaining codon positions (median = −0.1%; Wilcoxon rank-sum test, *p*-value = 0.003). There were only two known CTL epitopes in the HCV E2 HVR1 sequence, both classified as HLA-A–restricted. We found no significant association between the quantity *mN − mS* and codon positions located within these epitopes (Wilcoxon rank-sum test, *p*-value = 0.87). In sum, nonsynonymous mixtures tend to accumulate disproportionately at codon positions under CTL selection, preferentially within HLA-B–restricted epitopes.

### Simulation Results

**Figure 2:**
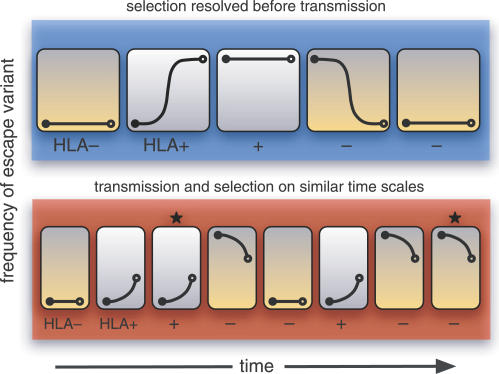
Effect of Transmission Rate on the Frequency of Mixtures This schematic depicts the transmission chain of a virus population, where each host is represented by an enclosed graph that represents the evolving frequency of a CTL escape variant over time. The hosts either possess an HLA allele which favors the escape variant (HLA^+^, orange-shaded boxes) or the wild-type virus (HLA^−^, white-shaded boxes). A severe transmission bottleneck causes the population in the next host to be initially fixed for either the wild-type or escape variant (filled circle). If selection for escape or reversion is sufficiently strong (upper schematic in blue), then the favored virus genotype will tend to become fixed within the host before transmission occurs (open circle). Under such conditions, transient polymorphisms will only occur whenever the virus is transmitted between hosts of opposite type. On the other hand, if transmission and selection occur on similar time scales (lower schematic in red), then the host type does not necessarily predict which virus genotype becomes transmitted, causing transient polymorphisms to become more abundant (starred boxes).

A surplus of nonsynonymous mixtures within CTL epitopes represents transient polymorphisms that are eventually driven to fixation in the host by selection for escape or reversion [28]. This implies that the probability of sampling nonsynonymous sequencing mixture should decline with the intensity of host-specific selection at that codon position. As a result, host-specific selection would produce negative correlation between *mN − mS* and *dN − dS* across codon positions in the range *dN − dS* > 0, contrary to what we have observed in HIV-1 and HCV gene sequences. This paradox can be reconciled by incorporating the early transmission of unfavorable variants into a model of virus evolution (Figure 2). When selection and transmission act on similar time scales, the composition of the circulating virus population (i.e., the source of new infections) will not necessarily match the diversity of HLA genotypes in the host population. Suppose that an escape variant is transmitted from a host with a rare HLA genotype to a new host with a common HLA genotype. If the escape variant cannot outcompete the wild-type virus in the absence of a CTL response, then selection will favor reversion [7,8]. But the selective advantage of the wild-type virus may be so narrow that a substantial probability remains of transmitting the original escape variant [8,14]. Under such conditions, the severe bottleneck upon transmission could fix either the wild-type or escape variant in the new individual population (Figure 2). Because the next host will likely have the common HLA genotype, this transmission event can recreate the selective conditions requiring a transient nonsynonymous polymorphism to occur.

**Figure 3:**
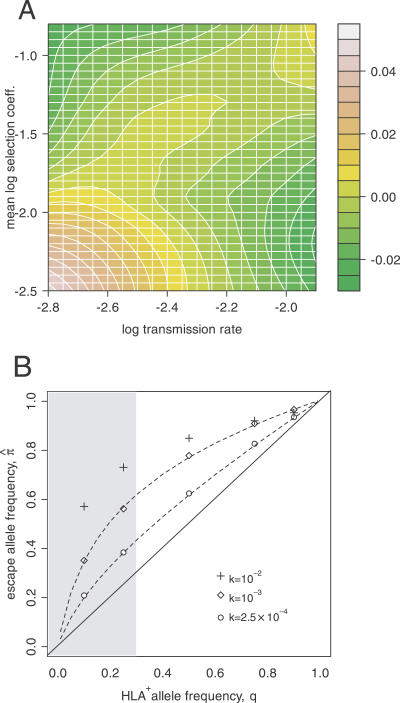
Factors Influencing Within-Host Polymorphisms and the Global Frequency of Escape Variants (A) A contour plot depicting the mean effect of selection and transmission rate on displacing the frequency of detectable polymorphisms from the neutral expectation (Δ*f_poly_*, refer to the color key), as estimated from simulations. (The expectation E[*f_poly_*] is jointly determined by the forward and back mutation rates, μ and ν, and population size, *N*.) The *x*-axis corresponds to the log-transformed transmission rate, log_10_ *k*. The *y*-axis represents the mean log-transformed selection coefficient, *E*(log_10_ *s*) = *q*log_10_(*s_esc_)* + (1 − *q*)log_10_(*s_rev_)*. (B) A 10-fold disparity in selection intensities *s_esc_* = 0.02, *s_rev_* = 0.002) causes π˄ to substantially exceed *q* with increasing transmission rate, *k*. Each set of points represents mean estimates of π˄ from simulations (with virus population size *N* = 5,000 and μ = ν = 10^−4^). Dashed lines indicate predicted values from the deterministic model, which performs poorly when *k* is too high (i.e., when transmissions occur rapidly, allele frequencies are almost always near zero or one where stochastic variation is greatest [31]). The typical range of *q* is indicated by the shaded plot region.

To investigate this hypothesis, we implemented a simulation of allele frequency evolution within individual virus populations with ongoing transmission through a succession of hosts. Each individual virus population was represented by a single locus containing either an escape variant (at frequency *p*) or the wild-type allele. We assumed that transmission of the virus to a new host involved a severe bottleneck, such that the next population was initially fixed for either the escape variant (with probability *p*) or wild-type allele. Viral fitness in a given host was determined by a single MHC locus, at which an allele restricting the wild-type virus (HLA^+^) was present at a frequency *q* in the host population. We observed that the mean frequency of within-host polymorphisms *f_poly_*:0.2 ≤ *p* ≤ 0.8 converged over time to an equilibrium value, which declined with stronger host-specific selection if the transmission rate was low (Figure 3A). On the other hand, if the transmission rate was high, then *f_poly_* increased with stronger selection and thereby became positively correlated with genetic variation among hosts.

By sustaining high levels of polymorphism within hosts, a joint increase in selection and transmission rate may also cause the frequency of the escape mutation in the circulating virus population (π = *E*(*p*)) to depart substantially from the expected value at equilibrium in the absence of polymorphism ( π˄ = *q,* i.e., individual virus populations fix alleles matching host HLA genotypes). In our simulations, if selection favoring escape in HLA^+^ hosts was sufficiently stronger than selection for reversion in HLA^−^ hosts, then π˄ became substantially greater than *q* at equilibrium (Figure 3B). On the other hand, if selection favoring reversion in HLA^−^ hosts was greater, then the equilibrium value of π˄ was deflected in the opposite direction, below *q* (not shown). This departure of π˄ from *q* became more pronounced with increasing transmission rates. Unequal mutation rates between the virus alleles could also contribute to this effect (Figure S3). An escape allele may therefore predominate the circulating virus population even when the selective HLA allele in the host population is rare. In other words, an individual possessing a rare HLA allele may nevertheless stand a high chance of becoming infected by a matched escape variant if selection for reversion is weak and the transmission rate is high.

### Deterministic Model of Viral Evolution

This process sustaining high levels of nonsynonymous polymorphism at codon positions under host-specific selection is related to the maintenance of genetic variation in a subdivided population by local adaptation [29,30] and can be illustrated with a simple deterministic model. We use the following differential equation [31]:

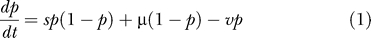
to describe the mean rate of change in *p* within a given host, where *s* is the selection coefficient, and μ and ν are the forward and back mutation rates, respectively. Initial conditions for Equation 1 were defined to reflect the severe bottleneck imposed by transmission of the virus (i.e., *p*(0) = 0 or *p*(0) = 1). Assuming that transmission occurs after a constant time interval (τ), the expected value of π after *n* transmissions is obtained from the recurrence equation:

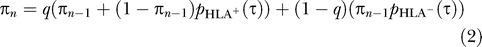
where *p*_HLA^+^_ and *p*_HLA^−^_ are approximate solutions of Equation 1 for evolution of *p* in HLA^+^ and HLA^−^ hosts, respectively (Protocol S1). Equation 2 has an equilibrium solution:

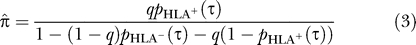
which reduces to π˄ = *q* when μ = ν and selection for escape and reversion is symmetric between host types (*s_esc_* = *s_rev_*). As τ approaches ∞, π˄ also converges towards *q* because the evolution of the escape allele within hosts is resolved before transmission (i.e., 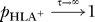 and 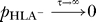 ). Conversely, as τ approaches zero, π˄ converges towards a quantity determined by the ratio of ν and μ (Protocol S2). The behavior of π˄ at these limits implies the existence of an intermediate waiting time to transmission (τ*_max_*), which maximizes the departure of π˄ from *q*. An approximation of τ*_max_* indicates that it is on the order of max(*s_esc_*,*s_rev_*)^−1^ when selection is stronger than mutation (Protocol S3). Thus, our model confirms that the greatest departure of π˄ from the expectation *q* occurs when the mean transmission rate corresponds to the overall intensity of selection.

We found a strong correspondence between this model and simulations (Pearson's ρ = 0.92, *p*-value < 10^−15^; Figure S4) with all incongruous cases being caused by stochastic effects due to effective population sizes within hosts of *N* = 10^2^ or below. The effective population size for HIV-1 is estimated to be on the order of 10^3^ and greater, while the total census population size is typically several orders of magnitude larger [32–34], and the census size for HCV is approximately 10-fold greater still. Hence, this model is a reasonably accurate representation of evolution within realistic HIV-1 and HCV populations.

## Discussion

In this study, we have described a novel pattern in the genetic variation of two human viruses, and formulated a simple population genetic model, supplemented with stochastic simulations, to explain it. However, because of the limited availability of population-based sequences that have not been stripped of sequencing mixtures, we were required to restrict our analysis to the RT and PR coding region of HIV-1, in which mixtures provide useful information on the evolution of resistance [21]. Although we focused our investigation on subtype B sequences isolated from treatment-naive individuals, we had no direct control over the sequencing and base-calling conditions of this data set. On the other hand, we obtained unprocessed sequencing electropherogram data of the HCV E1 envelope coding region, such that we could uniformly apply our own methods across all sequences. We were also unable to control for the circumstances under which sequences were isolated from either HIV-1– or HCV-infected patients, e.g., days since infection or seroconversion, regionality of patient populations. Even so, these sampling issues would not bias inferences based on site-by-site comparisons of sequence variation (e.g., relative mixture frequencies). We were able to recover an exceptionally clear and consistent signal of a link between within-host and among-host genetic variation among codon positions in HIV-1 and HCV sequences. This pattern represents strong evidence for CTL-mediated selection in both viruses, specifically targeting with HLA-B–restricted epitopes.

The rapid accumulation of genetic variation in HIV-1 and HCV enables these viruses to elude the immune system and forestalls the development of effective vaccines. Identifying the factors that shape genetic diversity in these human viruses remains a formidable challenge. Because these viruses possess exceptionally high mutation rates, extensive genetic variation accumulates within hosts that may be shaped by ongoing host-specific adaptation. However, the development of models of virus evolution within hosts has been largely independent of “dynamical” models of the transmission and spread of viruses across host cells and individuals [25]. As a result, few models of virus evolution integrate the evolution within hosts with viral dynamics at the level of the host population, which could otherwise reveal emergent properties of evolution within hosts. For example, there is an extensive literature characterizing selection in HIV-1 [10,35–47] by comparing inferred rates of nonsynonymous and synonymous substitutions, but these studies employ methods that do not explicitly distinguish between within- and among-host variation (but see [19,48]).

However, empirical evidence indicates that aspects of the host population can influence patterns of evolution within hosts, and vice versa. For instance, Ross and Rodrigo [10] found evidence that the magnitude and persistence of site-specific diversifying selection within patients was correlated with the rate of progression to acquired immune deficiency syndrome (AIDS), which may influence long-term epidemiological dynamics in the host population. Moore et al. [9] found significant associations between divergent codon positions within CTL epitopes in HIV-1 RT and HLA allelic variation in the host population, which implied that CTL-mediated selection within hosts was influencing the evolution of the total virus population. More recently, Kosakovsky Pond et al. [48] developed a customized phylogenetic analysis to detect significant turnover in codon positions under diversifying selection in HIV-1 PR and RT sequences among human populations with distinct HLA frequencies. They also found that many nonsynonymous substitutions that were mapped to terminal branches of the tree (i.e., occurring within hosts) were absent from internal branches, suggesting that adaptations within individual virus populations were not always maintained at the level of the total virus population [48].

These observations motivate the theoretical development of models of viral evolution that capture the interaction between the within-host and among-host levels of genetic variation. Recently, Grenfell et al. [24] sought to unify the characteristic shape of phylogenetic trees for different virus pathogens with the evolutionary processes within hosts. For instance, phylogenetic trees derived from HIV-1 or HCV sequences sampled from the host population tend to be more “balanced”, reflecting the epidemiological spread of the virus [24]. In contrast, trees derived from influenza A virus hemagglutinin sequences are less balanced, containing a persistent “backbone” that continually spawns short-lived lineages [49]. They proposed that this variation in tree shape, which indirectly manifests the genetic variation among hosts, was driven by the rate at which variants with a selective advantage in the previous host were being transmitted to the subsequent host. Our model complements this previous work by directly evaluating the influence of within-host evolution on the accumulation of nonsynonymous substitutions that differentiate individual virus populations, and the reciprocal effect of this divergence among hosts on variation within hosts. As a result, we can obtain quantitative predictions on how selection within hosts and the transmission rate will influence the frequency of escape variants in the total virus population. The model also predicts that variation in the mean surplus of nonsynonymous mixtures (quantified by the summary statistic *mN − mS*) per gene indicates divergent intensities of host-specific selection. Similarly, the characteristic transmission rates and overall intensity of selection of different viruses (e.g., HIV-1, HCV, influenza A virus) may revealed by a divergence in the mean surplus of nonsynonymous mixtures per virus. We did not attempt to infer differences between genes or viruses from the absolute frequencies of mixtures in the current data set due to the potential variation in sequencing protocols (as discussed above). Nevertheless, our model should motivate investigators in viral evolution to provide access to raw sequencing data, including annotation of variables that could influence the detection of polymorphisms (e.g., lab sequencing protocol, automated sequencer type and manufacturer).

Based on the distribution of relative mixture frequencies (i.e., site-by-site comparisons within genes), our model indicates that the genetic variation of HIV-1 and HCV is being shaped by the ongoing transmission of unfavorable variants, skewing the frequency of an escape variant in the total virus population towards the direction that host-specific selection is strongest. This unexplored imprint of within-host evolution, manifested as a site-specific surplus of nonsynonymous mixtures within CTL epitopes, can strongly influence the overall composition of the circulating virus population, in addition to founder effects. Because we observed this phenomenon in both HIV-1 and HCV, it may be a common feature of viruses that exhibit both prolific genetic variation within hosts and substantial rates of transmission.

## Materials and Methods

### HIV-1 and HCV sequence data.

We obtained treatment-naive HIV-1 subtype B sequences from the HIV Drug Resistance Database at Stanford University (Stanford HIVDB) [50]. At the time of analysis, there were 3,458 PR and 1,997 RT sequences meeting our criteria, representing 3,004 and 1,989 patients, respectively. By restricting the data set to treatment-naive individuals, we sought to minimize the confounding effects of selection for drug-resistant variants. Further screening for antiviral resistance was carried out by aligning each sequence to its closest subtype reference sequence (obtained from the Los Alamos National Laboratory [LANL] HIV sequence database; [51]) and scoring for resistance according to the Stanford HIVDB mutation scores using customized scripts in HyPhy [52]. Assuming worst-case resolution of ambiguous nucleotides (i.e., maximized scores), 149 RT and 58 PR sequences with at least low-level resistance (score ≥ 15) were discarded from the data sets. All 297 nucleotide sites from PR sequences were included in our analyses. RT sequences were truncated to nucleotide sites 1 to 741 to exclude poorly sampled tail regions from the analyses.

In addition, we obtained 2,691 chromatogram traces generated from ABI 310 and Beckman CEQ 8000 automated sequencers, covering the core E1 region of HCV. For the majority of traces, each corresponded to a unique isolate from a patient for the initial diagnosis and genotyping of an HCV infection. All trace files were converted to standard chromatogram format and processed with the base-calling program Phred [53]. Potential sequencing mixtures were identified by screening the uncalled peak output using a custom Python script. An uncalled peak was classified as representing a minority variant if: (1) it was located within ±1 trace points of a called peak; and the area under the uncalled peak was (2) at least 20% of the called peak area; (3) at least 10% the mean area of the last ten called peaks; and (4) at least two times greater than the mean area of the last five uncalled peaks. All sequences were truncated to the E1 coding region spanning the nucleotide sites 1 to 399. We also obtained 346 published population-based RT-PCR sequences from Genbank (see Accession Numbers) spanning the hyper-variable region HVR1 of HCV envelope protein E2 [54–57].

### Site-specific estimation of substitution rates.

Sequences were aligned using ClustalW [58] and manually adjusted with Se-Al version 2.0 [59] (alignments available upon request). We used neighbor-joining [60] with Tamura-Nei [61] distance to reconstruct the phylogeny from each sequence alignment. Pairwise distances from each phylogeny indicated that the sequences were highly divergent (Figure S5). To estimate the number of nonsynonymous and synonymous substitutions with branch corrections at each codon position, we employed the single-likelihood ancestor counting method [62] as implemented in HyPhy [52,63] using the default settings. Ambiguious nucleotides were resolved to the consensus codon at that position in order to remove any possible influence of mixture frequencies on estimates of substitution rates. We tested for significant positive selection *(dN* > *dS)* by applying a continuous extension of the binomial distribution to model the probability that a given proportion of substitutions are nonsynonymous, given the proportion of sites that are nonsynonymous at the codon position [63].

### Association with CTL epitopes.

For analyzing associations between nonsynonymous mixture frequencies and epitopes within HIV-1 PR and RT, we applied the CTL epitope definitions from the LANL HIV immunology database [64]. Similarly, we applied the CTL epitope definitions from the LANL HCV immunology database for analyzing associations within HCV E1 and E2 (HVR1) [65].

### Simulations of virus evolution.

We implemented a simulation of virus evolution in a host population using an iterative Moran process [66]. Both virus and host populations were each modeled by a single two-allele locus, representing the immune escape and HLA genotypes, respectively. Instantaneous rates for the unit increase and decrease of escape allele frequency within a host were:

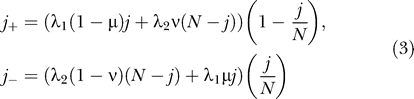
where *j* is the number of wild-type alleles in an ideal population of constant size *N,* and λ_1_ and λ_2_ are the wild-type and escape virus growth rates. If the host was HLA^−^, we set λ_1_ = 1 and λ_2_ such that the selection coefficient for reversion *s_rev_* = (λ_1_ − λ_2_)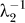 . Otherwise, we set λ_1_ < λ_2_ so that *s_esc_* = (λ_2_ − λ_1_)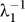 > 0. After an exponentially distributed waiting time (τ) with rate *k,* a randomly selected individual from *K* = 10^3^ hosts was replaced. This new host was HLA^+^ with probability *q* (and HLA^−^ otherwise), and infected by wild-type virus with probability *j*_τ_/*N,* where *j*_τ_ is obtained from the iterative application of *j*_+_ and *j*_−_, and the total number of events occurring in the time interval τ was determined by random draws from an exponential distribution with the rate (*j*_+_ + *j*_−_). Otherwise, it was infected by the escape mutant virus. This new infection was therefore initially fixed for either the wild-type or escape virus genotype, assuming a severe bottleneck upon transmission between hosts.

Simulations were run for 200 × *K* transmissions, which was sufficient for π to converge to an equilibrium for all parameter values evaluated. We recorded the frequency of the escape allele in the individual virus population (*p* = 1 − *j*/*N*), from which we calculated the mean frequency among hosts (π = *E*(*p*)). Given the empirical detection threshold of minority variants from population-based sequencing, an individual virus population was considered to be detectably polymorphic if 0.2 < *p* <0.8. Unique parameter values were assigned to 100 replicate simulations by Latin hypercube sampling from their respective ranges: *q* = (0, 0.5); μ,ν = (10^−5^, 10^−3^); *s_esc_*,*s_rev_* = (0.002, 0.2); *N* = (10^2^, 10^5^); and *k* = (0.00137, 0.0137), such that transmissions occur after approximately 0.2 to 2 years (where τ is in units of days).

To compare the simulation results to our deterministic model, we used the numerical integration function in *Mathematica* 5.1 (Wolfram Research, http://www.wolfram.com) to calculate the expectation of Equation 3 assuming that the waiting time τ was exponentially distributed with rate parameter *k*.

## Supporting Information

Figure S1 Histograms for the Frequency of Nonsynonymous and Synonymous Mixtures per Sequence The range of frequencies for HCV E2 (HVR1) has been truncated at ten mixtures per sequence for clarity, although a small number of sequences contain as many as 18 mixtures. In HIV-1 PR and RT and HCV E2 (HVR1), there is an excess of mixture-free sequences, possibly due to an under-reporting bias of mixtures which are often interpreted as sequencing errors. HCV E1 sequences were obtained directly from unprocessed trace files and were not subject to this bias. The level of dispersion in the observed frequency distributions was evaluated by fitting Poisson and negative binomial models using a generalized linear models procedure. Goodness-of-fit, quantified by Akaike's information criterion, was improved by the negative binomial model in all cases, and estimates of the dispersion parameter confirmed overdispersion of mixture frequencies in HIV-1 PR and RT and HCV E2 (HVR1).
(13 KB PDF)

Figure S2 Mixture Frequency Distributions The histograms depict frequency distributions for nonsynonymous (above) and synonymous (below) mixtures per codon position. Note that the histograms for HCV E1 and E2 (HVR1) are on different scales. There is conspicuously greater variation among codon positions in nonsynonymous mixture frequencies, more notably in HIV-1 sequences. Codon positions associated with peaks in the frequency of nonsynonymous mixtures are indicated above each distribution by the alignment consensus amino acid and residue number.
(169 KB PDF)

Figure S3 Effect of Mutation Rate Asymmetry on the Frequency of Escape Mutations A contour plot depicting the difference π˄ − *q* as a function of the disparity in selection coefficients and mutation rates between HLA^+^ and HLA^−^ hosts (*r* = log_10_(*s_esc_*) − log_10_(*s_rev_*) + log_10_μ − log_10_ν) and the transmission rate (log_10_ *k*). When the net effect of mutation and selection is equivalent between HLA^+^ and HLA^−^ hosts (*r* = 0), then π˄ converges to *q* and is independent of variation in transmission rate. In contrast, when there is a net imbalance in mutation and selection (*r* ≠ 0), there is a departure of π˄ from *q*; this departure becomes greater with increasing transmission rates. Viral population size has no apparent effect on the difference π˄ − *q*. Each open circle corresponds to a replicate simulation with unique parameter values set by Latin hypercube sampling.
(30 KB PDF)

Figure S4 Comparison of Stochastic Simulation and Model Predictions Scatterplot illustrating correspondence between predicted value of π from the deterministic model (*x*-axis) and values obtained from simulations at equilibrium ( π˄, *y*-axis). A solid line is drawn at *x* = *y* to indicate an exact match between model and simulation frequencies. Dashed lines above and below the *x* = *y* axis enclose variation in frequencies within a ±10% interval. Disparity between the model and simulations is caused by a lack of stochastic factors in the model. Replacing the unidirectional mutation approximations of the model (*p_HLA^+^_* and *p_HLA^−^_*) by the exact formula has no visible effect on the correspondence between the model and simulations.
(9 KB PDF)

Figure S5 Frequency Distributions of Pair-Wise Distances of HIV-1 and HCV Sequences There is a substantial amount of divergence among the vast majority of HIV-1 sequences in the reconstructed phylogenetic trees, with only <0.1% of pairwise distances below 0.01. This is consistent with the low number of HIV-1 PR and RT sequences that were re-sampled from the same patient. HCV E1 and E2 (HVR1) sequences were highly divergent on average. A small proportion of pairwise distances between HCV E1 sequences (1.1%), particularly in subtype 4d, were below 0.05. Similarly, about 3% of pairwise distances between HCV E2 (HVR1) sequences were below a threshold of 0.25. Hence, a minority of HCV sequences may have represented multiple isolates from patients, but were too few overall to influence the outcome of our analyses.
(14 KB PDF)

Protocol S1 Approximation of Allele Frequency Evolution (73 KB PDF)

Protocol S2 Limit Behavior of Deterministic Model (41 KB PDF)

Protocol S3 Approximation of Optimal Waiting Time to Transmission (60 KB PDF)

### Accession Numbers

GenBank (http://www.ncbi.nlm.nih.gov/Genbank/index.html) accession numbers for the HCV E1 envelope protein-coding sequences used in our study are AY766700–AY768365. GenBank accession numbers for the E2 envelope protein (HVR1) sequences used in our study are the following: AY390002, AY390005, AY390008, AY390010, AY390013, AY390016, AY390019, AY390022, AY390024, AY390027, AY390030, AY390032, AY742960–AY743049, AY309923–AY309954, AY314963–AY314969, AY390002–AY390035, AY564735–AY564784, and AY935999–AY936132.

## Abbreviations

CTL: cytotoxic T-lymphocyte
HCV: hepatitis C virus
HIV-1: human immunodeficiency virus type 1
HLA: human leukocyte antigen
PR: protease
RT: reverse transcriptase
