# Supplementary figures and images for: Adaptation to Human Populations Is Revealed by Within-Host Polymorphisms in HIV-1 and Hepatitis C Virus
Source: PLoS Pathog. 2007 Mar 30;3(3):e45. doi: 10.1371/journal.ppat.0030045 (PMC1839164; doi:10.1371/journal.ppat.0030045)

# HIV-1 PR

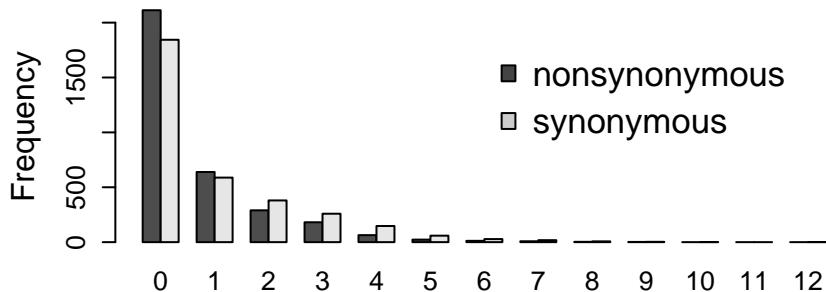

# HIV-1 RT

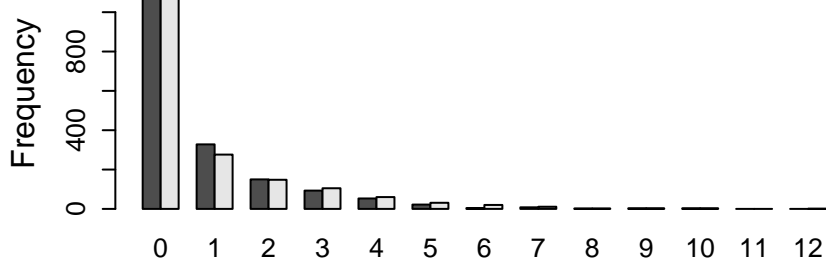

# HCV E1

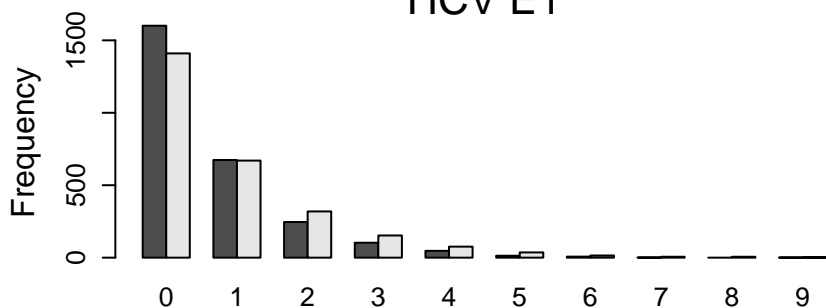

# HCV E2(HVR1)

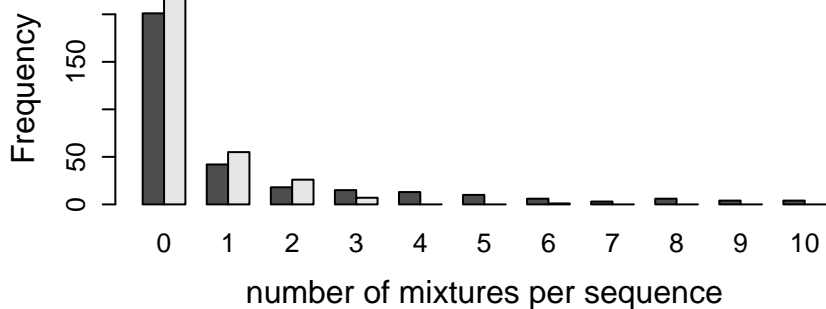

Supplement: Figure S1 — The range of frequencies for HCV E2 (HVR1) has been truncated at ten mixtures per sequence for clarity, although a small number of sequences contain as many as 18 mixtures. In HIV-1 PR and RT and HCV E2 (HVR1), there is an excess of mixture-free sequences, possibly due to an under-reporting bias of mixtures which are often interpreted as sequencing errors. HCV E1 sequences were obtained directly from unprocessed trace files and were not subject to this bias. The level of dispersion in the observed frequency distributions was evaluated by fitting Poisson and negative binomial models using a generalized linear models procedure. Goodness-of-fit, quantified by Akaike's information criterion, was improved by the negative binomial model in all cases, and estimates of the dispersion parameter confirmed overdispersion of mixture frequencies in HIV-1 PR and RT and HCV E2 (HVR1). (13 KB PDF) [file ppat.0030045.sg001.pdf]

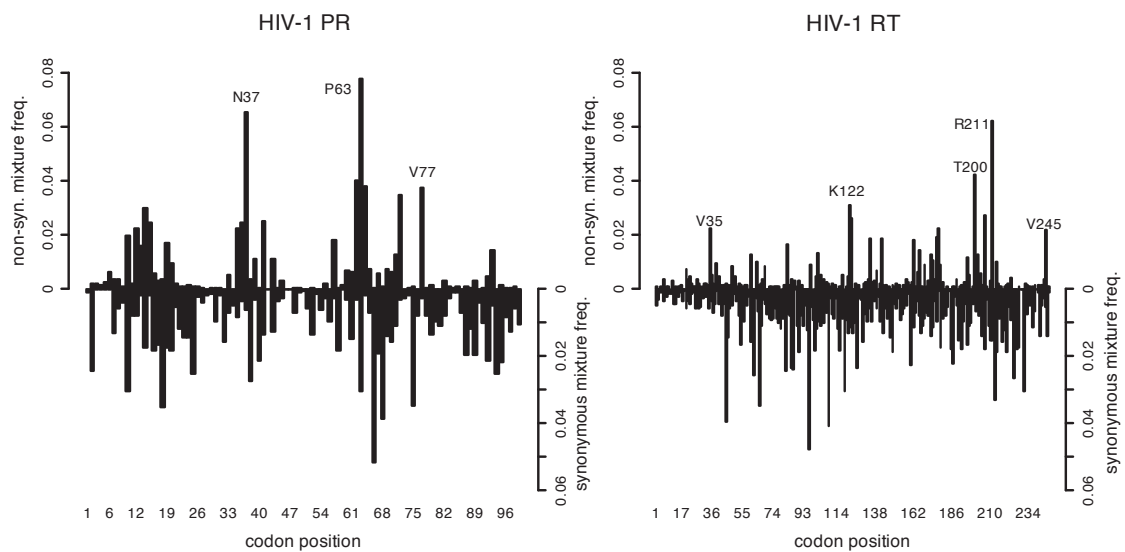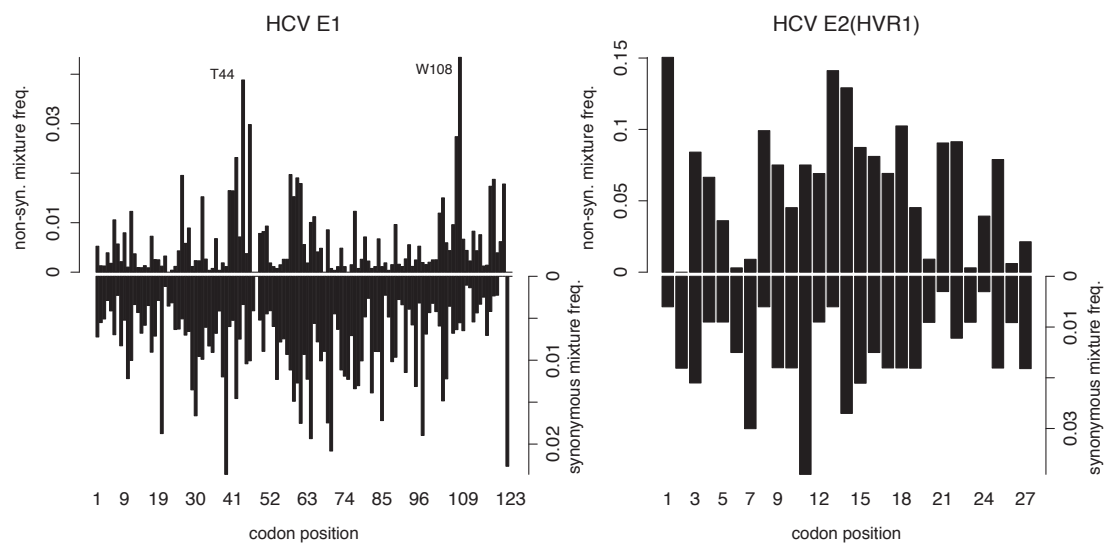

Supplement: Figure S2 — The histograms depict frequency distributions for nonsynonymous (above) and synonymous (below) mixtures per codon position. Note that the histograms for HCV E1 and E2 (HVR1) are on different scales. There is conspicuously greater variation among codon positions in nonsynonymous mixture frequencies, more notably in HIV-1 sequences. Codon positions associated with peaks in the frequency of nonsynonymous mixtures are indicated above each distribution by the alignment consensus amino acid and residue number. (169 KB PDF) [file ppat.0030045.sg002.pdf]

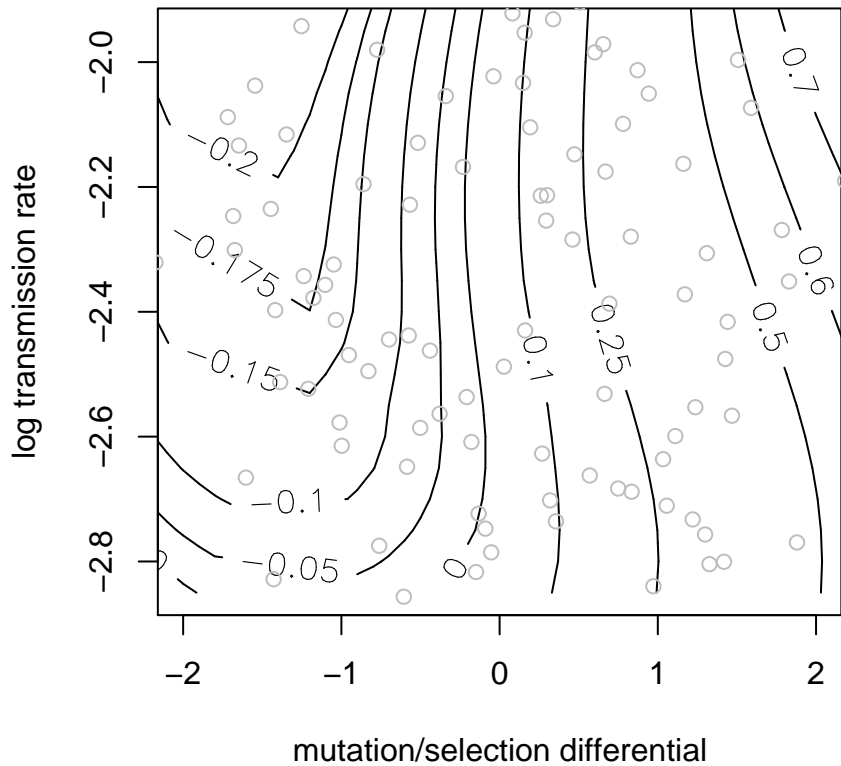

Supplement: Figure S3 — A contour plot depicting the difference π˄ − q as a function of the disparity in selection coefficients and mutation rates between HLA+ and HLA− hosts (r = log10(sesc) − log10(srev) + log10μ − log10ν) and the transmission rate (log10 k). When the net effect of mutation and selection is equivalent between HLA+ and HLA− hosts (r = 0), then π˄ converges to q and is independent of variation in transmission rate. In contrast, when there is a net imbalance in mutation and selection (r ≠ 0), there is a departure of π˄ from q; this departure becomes greater with increasing transmission rates. Viral population size has no apparent effect on the difference π˄ − q. Each open circle corresponds to a replicate simulation with unique parameter values set by Latin hypercube sampling. (30 KB PDF) [file ppat.0030045.sg003.pdf]

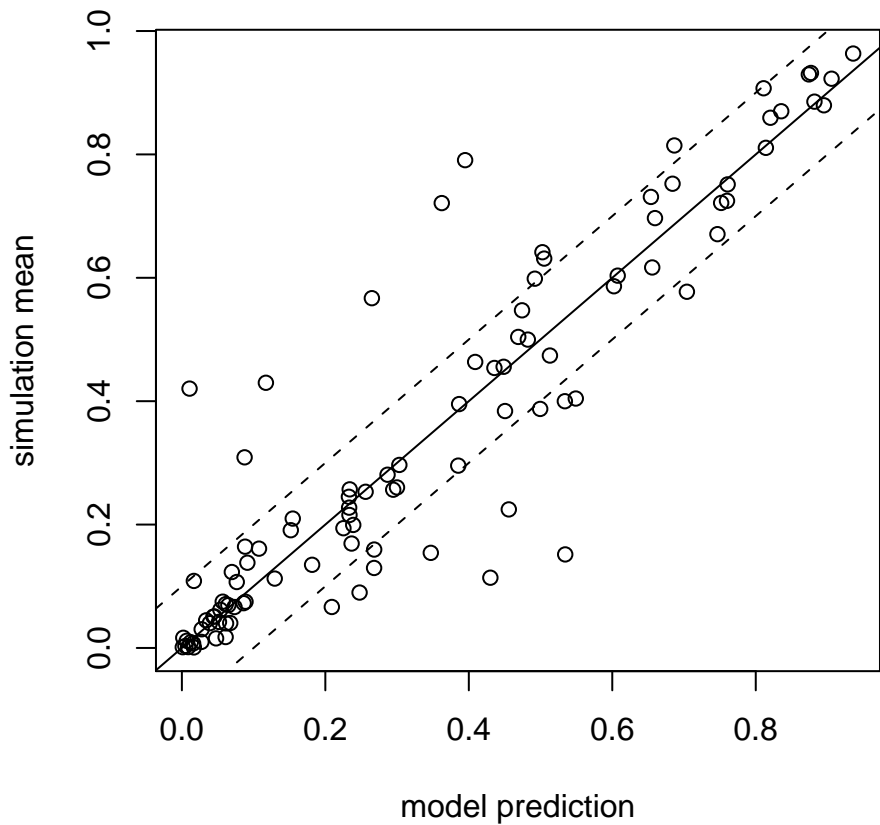

Supplement: Figure S4 — Scatterplot illustrating correspondence between predicted value of π from the deterministic model (x-axis) and values obtained from simulations at equilibrium ( π˄, y-axis). A solid line is drawn at x = y to indicate an exact match between model and simulation frequencies. Dashed lines above and below the x = y axis enclose variation in frequencies within a ±10% interval. Disparity between the model and simulations is caused by a lack of stochastic factors in the model. Replacing the unidirectional mutation approximations of the model (pHLA+ and pHLA−) by the exact formula has no visible effect on the correspondence between the model and simulations. (9 KB PDF) [file ppat.0030045.sg004.pdf]

# HIV-1 PR

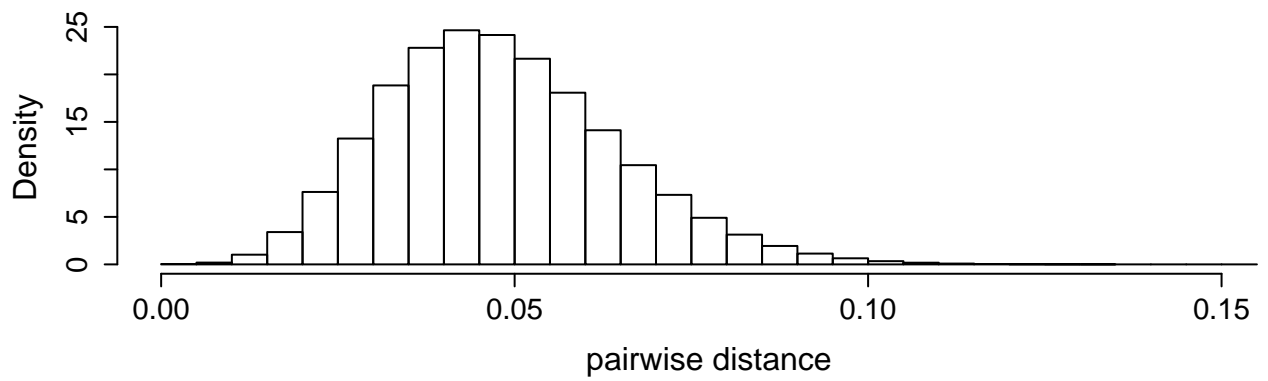

# HIV-1 RT

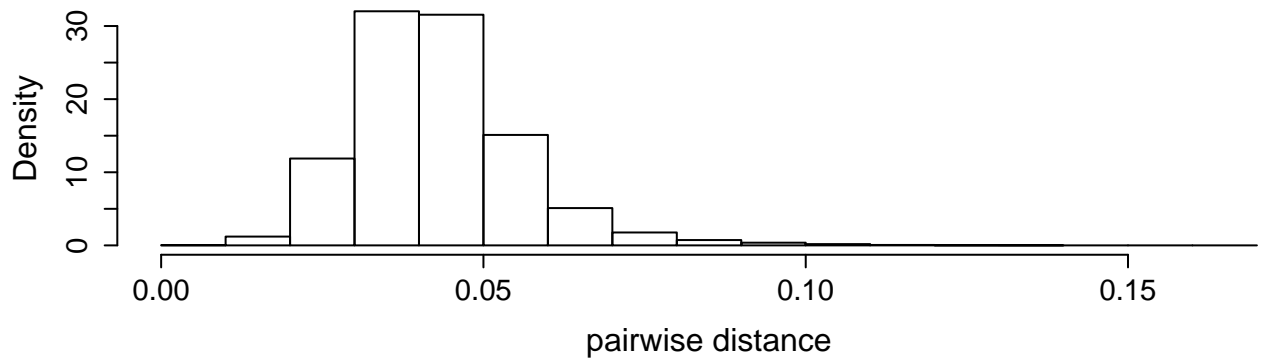

# HCV E1

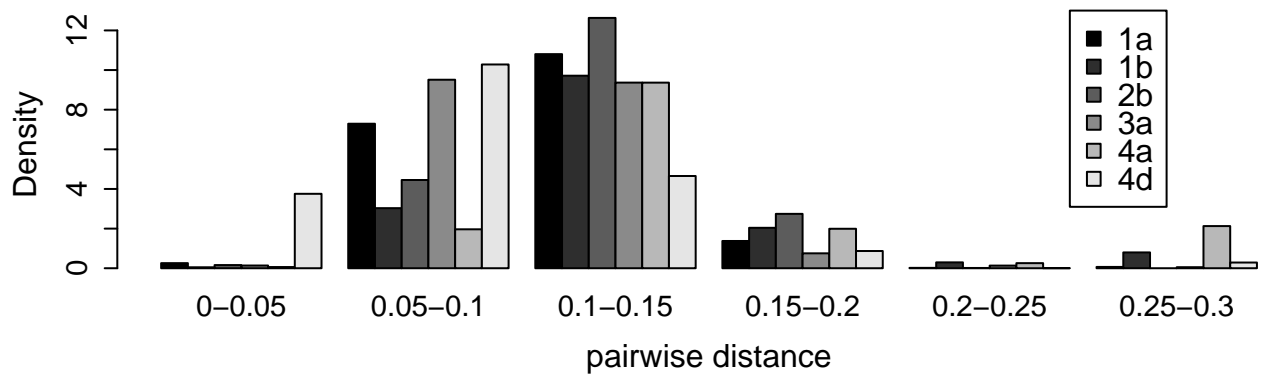

# HCV E2 (HVR1)

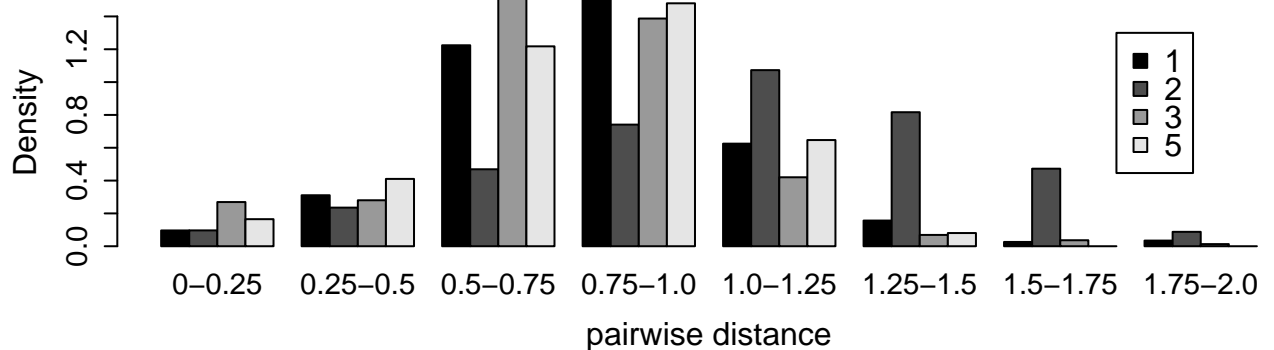

Supplement: Figure S5 — There is a substantial amount of divergence among the vast majority of HIV-1 sequences in the reconstructed phylogenetic trees, with only <0.1% of pairwise distances below 0.01. This is consistent with the low number of HIV-1 PR and RT sequences that were re-sampled from the same patient. HCV E1 and E2 (HVR1) sequences were highly divergent on average. A small proportion of pairwise distances between HCV E1 sequences (1.1%), particularly in subtype 4d, were below 0.05. Similarly, about 3% of pairwise distances between HCV E2 (HVR1) sequences were below a threshold of 0.25. Hence, a minority of HCV sequences may have represented multiple isolates from patients, but were too few overall to influence the outcome of our analyses. (14 KB PDF) [file ppat.0030045.sg005.pdf]
