# Supplementary material for: Adaptation to Human Populations Is Revealed by Within-Host Polymorphisms in HIV-1 and Hepatitis C Virus
Source: PLoS Pathog. 2007 Mar 30;3(3):e45. doi: 10.1371/journal.ppat.0030045 (PMC1839164; doi:10.1371/journal.ppat.0030045)
Supplement: Protocol S1 — (73 KB PDF) [file ppat.0030045.sd001.pdf]

Protocol S1. Approximation of allele frequency evolution.

To obtain an approximate solution of equation (3), we derive two approximate solutions of equation (1) in which mutation is assumed to be unidirectional with host-specific selection, giving:

$$p_{\mathrm{HLA}^+}(t) = \frac{\mu\left(e^{t(s_{esc}+\mu)} - 1\right)}{s_{esc} + \mu e^{t(s_{esc}+\mu)}}$$

and

$$p_{\mathrm{HLA}^-}(t) = \frac{s_{rev} + \nu}{s_{rev} + \nu e^{t(s_{rev}+\nu)}}.$$

These approximations have the initial conditions $p_{\mathrm{HLA}^+}(0) = 0$ and $p_{\mathrm{HLA}^-}(0) = 1$ and are reasonably accurate when selection is stronger than mutation and the effective population size is large.
