# Supplementary material for: Adaptation to Human Populations Is Revealed by Within-Host Polymorphisms in HIV-1 and Hepatitis C Virus
Source: PLoS Pathog. 2007 Mar 30;3(3):e45. doi: 10.1371/journal.ppat.0030045 (PMC1839164; doi:10.1371/journal.ppat.0030045)
Supplement: Protocol S2 — (41 KB PDF) [file ppat.0030045.sd002.pdf]

Protocol S2. Limit behavior of deterministic model.

To evaluate the limit of equation (3) as τ approaches 0, we apply a series expansion at $\tau=0$ to the unidirectional-mutation approximations to yield:

$$\hat{\pi} \xrightarrow{\tau\to 0} \left(1+\frac{1-q}{q}\frac{\nu}{\mu}\right)^{-1}.$$

The departure of this limit from q is determined by the ratio ν/μ. The first derivative of $\hat{\pi}$ with respect to τ as $\tau\varnothing 0$ is approximated by the formula:

$$\left.\frac{\partial\hat{\pi}}{\partial\tau}\right|_{\tau=0} = \frac{q(1-q)\mu\nu(s_{esc}+\mu-s_{rev}-\nu)}{2(q(\mu-\nu)+\nu)^2}+O(\tau^2).$$

This quantity is greater than zero when $s_{esc}+\mu>s_{rev}+\nu$ (*i.e.* when the net effect of selection and mutation is biased towards the escape mutant allele).
