# Supplementary material for: Adaptation to Human Populations Is Revealed by Within-Host Polymorphisms in HIV-1 and Hepatitis C Virus
Source: PLoS Pathog. 2007 Mar 30;3(3):e45. doi: 10.1371/journal.ppat.0030045 (PMC1839164; doi:10.1371/journal.ppat.0030045)
Supplement: Protocol S3 — (60 KB PDF) [file ppat.0030045.sd003.pdf]

Protocol S3. Approximation of optimal waiting time to transmission.

Taking the first derivative of equation (3) leads to a complicated expression, which can be simplified by removing terms of order $O(e^{-\tau})$, where τ is on the order 10^2 or more, giving:

$$\tau_{max} \approx \ln\left(\frac{\mu}{\nu}\right) + 2\frac{\ln(s_{esc} + \mu) - \ln(s_{rev} + \nu)}{(s_{esc} + \mu) - (s_{rev} + \nu)}.$$

Compared to numerical root-finding procedures, this approximation yields reasonably accurate estimates within 10% of the true value over a broad range of parameter values.
